# A Label-Free Cell-Based Biosensor Method for Ethanol Quantification Using Temperature-Induced Spontaneous Cell Detachment

**DOI:** 10.3390/bios16070355

**Published:** 2026-06-25

**Authors:** Derick Yongabi, Alex Krane, Heloisa Espreafico Guelerman Ramos, Sofia Xavier Bustia, Jonas Gruber, Michael J. Schöning, Frank Delvigne, Patrick Wagner

**Affiliations:** 1 Laboratory for Soft Matter and Biophysics, Department of Physics and Astronomy, KU Leuven, Celestijnenlaan 200 D, 3001 Leuven, Belgium; alex.krane@aphea.bio (A.K.); patrickhermann.wagner@kuleuven.be (P.W.); 2 Faculdade de Ciências Farmacêuticas, Universidade de São Paulo, Av. Prof. Lineu Prestes, 580, São Paulo CEP 05508-000, SP, Brazil; heloisaramos139@usp.br (H.E.G.R.); sofiabustia@usp.br (S.X.B.); 3 Instituto de Química, Universidade de São Paulo, Av. Prof. Lineu Prestes, 748, São Paulo CEP 05508-000, SP, Brazil; jogruber@iq.usp.br; 4 Institute of Nano- and Biotechnologies (INB), Aachen University of Applied Sciences, Heinrich-Mußmann-Straße1, D-52428 Jülich, Germany; schoening@fh-aachen.de; 5 Microbial Processes and Interactions (MiPI), Terra Research and Teaching Centre, Gembloux Agro-Bio Tech, University of Liège, Av. De la Faculté D’Agronomie 41/13, 5030 Gembloux, Belgium; f.delvigne@uliege.be

**Keywords:** ethanol quantification, heat transfer method, spontaneous cell detachment, cell quality monitoring, pharmaceutical drug screening, cell-based biosensor

## Abstract

Rapid, low-cost ethanol quantification is vital for beverage quality control, biofuel production, and pharmaceutical applications, yet current approaches are costly, reagent- or label-dependent, or rely on spectroscopy with substantial sample preparation. We introduce a purely cell-based, label-free biosensor that exploits temperature-gradient-induced spontaneous detachment of *Saccharomyces cerevisiae* from a chip surface. The readout is the detachment half-time, *t_d_*_50_, derived from time-resolved changes in interfacial thermal resistance, *R_th_*, at the solid–liquid interface. Cells were pre-exposed to ethanol (0–70% *v*/*v*) and the detachment kinetics monitored using the heat transfer method (HTM). Under these conditions, cells display a pronounced non-monotonic *t_d_*_50_ response with a peak around 20% *v*/*v* ethanol. Overall, the *t_d_*_50_ rises from ~45 min (0% ethanol) to ≳10 h (20%) and then decreases, with no detachment at 60% and beyond. Critically, cell quality gates the detachment window. Fresh yeast responds up to ~50%, whereas aged yeast ceases to detach above ~8%, demonstrating a dual-function assay. Complementary measurements show that ethanol decreases surface tension monotonically, as expected, while optical/SEM imaging reveals aggregation above the detachment window. Requiring only a heater and a temperature probe, this platform offers a compact and low-cost strategy for ethanol sensing. Its applicability in a complex matrix is further demonstrated using whiskey diluted to selected alcohol concentrations, which produced responses consistent with the ethanol calibration trend. Potentially, it also offers a thermal assay for real-time monitoring of microbial cell quality across biotechnology and bioengineering applications. Considering ethanol as a proxy for drugs, the strategy may also support label-free drug screening on cells. At a fundamental level, the non-monotonic effect of ethanol, and especially the sharp maximum at 20%, remains unresolved and invites further studies.

## 1. Introduction

Ethanol quantification is important for various industrial and research applications. For instance, in the brewing and winemaking industry, where ethanol is the major product, accurate ethanol measurement is required for quality control and regulatory compliance [1]. Extending beyond fermented beverages, precise ethanol quantification in distilled spirits, such as whisky, rum, vodka, and gin, is vital for process control, ABV (alcohol by volume) labeling/taxation, and authenticity checks [2]. Similarly, sensitive and reliable monitoring of ethanol levels plays a key role in maximizing fermentation yields for biofuels and ensuring accurate tracking of ethanol content in fuel blends [3,4]. In addition, it enables clinical and forensic determination of blood alcohol levels, as well as proper solvent content in products like pharmaceuticals, cosmetics, paints, and varnishes [4]. Therefore, analytical techniques that are fast, cost-effective, and sensitive, ideally providing real-time feedback for process control, are required. A variety of methods exist for ethanol quantification. For example, gas chromatography (GC) with flame ionization detection is a gold-standard laboratory method renowned for its accuracy [5]. Also, high-performance liquid chromatography (HPLC) and other classical bench assays, such as refractometry and spectrophotometry, are widely employed in routine analyses [1]. Spectroscopic approaches, such as infrared-based sensors, enable non-invasive ethanol measurements in certain contexts [6], while numerous enzymatic assays or biosensors have been developed using alcohol dehydrogenase or alcohol oxidase to achieve highly specific ethanol detection [1]. In addition, semiconductor gas sensors have been widely employed for online and real-time monitoring of ethanol production during fermentation, providing a complementary approach for tracking yeast metabolic activity in biotechnological processes [7]. However, each of these techniques has inherent limitations, thus necessitating the need for alternative methods. For instance, traditional chromatographic and spectroscopic methods, while accurate, rely on expensive instrumentation and skilled personnel and often require tedious sample preparation [1].

In the case of biosensor-based approaches, bioreceptors (enzymes or antibodies) are often used, which must be immobilized and coupled with other reagents, adding cost and complexity [8]. Specifically, enzyme-based ethanol sensors can suffer from limited stability (due to gradual enzyme denaturation) and interference from other substances in the samples, which restricts their reliability and reusability over time [8]. Other advanced assays also involve labeling steps or specialized materials, such as fluorescent probe indicators or nanostructured interfaces [1,8]. Moreover, some standard methods operate offline in centralized labs, providing delayed results and low throughput, which is incompatible with real-time process control needs [1]. These shortcomings highlight the need for easy alternative strategies that are low-cost label- and receptor-free while capable of real-time measurements with minimal sample preparation.

In this study, we aim to develop a multifunctional technique that goes beyond simple ethanol quantification to include applications in quality monitoring of cells, as well as serving as a cell-based pharmaceutical screening tool. For the specific case of yeast, ensuring its quality is critical because cellular state directly determines process performance, product consistency, and yield across baking, brewing, winemaking, bioethanol fuel, as well as yeast strain development [9,10]. In baking, for instance, yeast vitality and osmo-/freeze-tolerance govern CO_2_ evolution, with elevated sugar/osmotic load slowing fermentation and gas release [11]. In brewing and winemaking, inoculum quality shapes attenuation and off-flavor control, as well as aroma and acidity outcomes, linking yeast physiological status to product quality and reproducibility [12,13,14]. In bioethanol production, robustness under very-high-gravity conditions (high sugar or fermentable substrates) and tolerance to process inhibitors and ethanol limit productivity and stability [15,16]. Finally, modern strain development hinges on screening and selecting superior or ethanol-tolerant yeasts tailored to brewery, winery, and industrial contexts [15,17].

Current assessment methods for cell quality employ microscopy/hemocytometer counts alongside multiparameter flow cytometry with membrane integrity dyes, as well as metabolic dyes, to quantify viability, vitality, and population heterogeneity [18,19]. Physiological performance is tracked via fermentative capacity and attenuation tests (e.g., pressure/CO_2_ evolution, vitality assays) that predict brewing endpoints, such as diacetyl reduction [20,21,22]. Stress tolerance is often inferred from storage metabolite and membrane composition metrics (trehalose/glycogen; sterol/lipid remodeling) that correlate with survival under ethanol and other stresses [23,24]. Cell surface interaction traits are assessed using flocculation/sedimentation indices and label-free interfacial sensing (e.g., quartz crystal microbalance with dissipation, QCM-D) to capture adhesion and viscoelastic fingerprints [25,26]. Rapid, non-destructive spectral fingerprints (Fourier transform infrared spectroscopy, FTIR/Raman) also support species/strain discrimination and phenotyping [27,28]. While powerful, many of these methods depend on labels/receptors or substantial sample preparation and instrument time, which can potentially introduce assay-dependent biases [29,30,31,32]. Therefore, avoiding exogenous probes or labels is essential to minimize perturbations to intrinsic cellular behavior. Because ethanol is a stressor that disrupts yeast membranes and compromises viability, integrating ethanol quantification with a simultaneous, label-free cell quality readout is highly advantageous [24,33].

Finally, in the field of pharmaceutical drug development, cell-based assays are indispensable for screening drug candidates, typically employing methods such as cell viability tests (e.g., metabolic MTT or ATP-luminescence) [34], fluorescence-based reporter or cytotoxicity assays, and microscopy. These techniques also suffer from similar drawbacks related to labeling, cost, and acquisition of real-time information [35]. We present a versatile, cell-based, label-free assay for ethanol quantification that requires only a mild temperature gradient and standard temperature sensors. Treating ethanol as a pharmacological stressor, we further establish this approach as a potential strategy for label-free drug screening. The readout exploits the phenomenon of spontaneous, collective cell detachment from a solid chip under a thermal gradient, which we first reported in [36]. Our study showed that the detachment kinetics, particularly the detachment time, constitutes cell-specific “fingerprints” capable of discriminating among yeast and human cancer cell lines (MCF-7 and HeLa) and sensitive to biological and physicochemical factors, including co-cultures [36,37,38]. In this study, we aim to demonstrate that by probing how living cells respond to a controlled heat gradient, the proposed assay can measure direct dosing effects (ethanol or drugs) while concurrently providing valuable information about cell quality.

## 2. Materials and Methods

### 2.1. Preparation of Cell Suspensions and Od600 Measurements

All measurements were performed using Dr. Oetker (Machelen, Belgium) *S. cerevisiae* yeast, which is commercially available in dry aggregates. Cell suspensions were prepared by adding dry yeast aggregates to the ethanol–water mixture (deionized MilliQ water) with the desired ethanol concentration (10 mg yeast/mL) and mildly vortexed (500 rpm, 1 min) until the uniform suspension was achieved. This resulted in an optical density (OD) of ≈1.8 ± 0.2 at 600 nm wavelength. OD600 measurements were performed on 100× diluted samples using an Ultrospec 2100 pro from Biochrom Ltd. (Cambridge, UK). To demonstrate practical application in a complex matrix, the same protocol was used to prepare yeast suspensions in Jack Daniel’s Tennessee Whiskey (Jack Daniel Distillery, Lynchburg, TN, USA) diluted with Milli-Q water to different alcohol volume concentrations. To test the sedimentation of the cells, OD600 measurements of suspensions with different ethanol percentages were performed. A fast decrease in optical density means the cells sediment rapidly. For high ethanol concentrations, the dry aggregates of yeast are difficult to suspend. Therefore, to prepare cell suspensions with high levels of ethanol > 20%, the cells were first quickly suspended in MQ water before the right volume fraction of ethanol was added and vortexted again. Two categories of yeast cells were used: (1) aged yeast, 6 months after the expiry date, and additionally exposed to air at room temperature for 1 month, and (2) fresh yeast, which was unopened and in-date prior to measurement. Analysis of the metabolic activity of the two categories of cells is described later. Aged yeast cells were used as a practical model of a low-quality cell population resulting from storage beyond the manufacturer’s expiry date to evaluate whether the method could distinguish fresh yeast from cells with reduced metabolic activity and compromised cellular vitality. This is because in real-world applications, reduced cell quality often arises from inappropriate storage, aging, expiry, or contamination.

### 2.2. Surface Preparation

Unless otherwise stated, all measurements were performed on polyurethane-coated aluminium chips to control for biocompatibility effects. The polymer solution was prepared from a mixture of the initiator (4,4′- diisocyanatodiphenylmethane, 122 mg), with the monomer (bisphenol A, 222 mg), and crosslinker (phloroglucinol, 25 mg) as described in references [39,40,41,42]. Pre-polymerization was achieved at 65 °C for 200 min until gelling point. All reagents were used as provided by the supplier (Sigma Aldrich N.V., Diegem, Belgium) with a purity of ≥99.9%. A polymer layer (≈1.2 µm) was then deposited on 10 mm by 10 mm aluminium chips (Brico N.V., Korbeek-Lo, Belgium) by spin-coating (60 s, 2000 rpm, and 1000 rpm/s acceleration). Highly hydrophilic glass chips were prepared by exposing 10 mm by 10 mm substrates (Glaswarenfabrik Karl Hecht GmbH & Co, Sondheim, Germany) to UV ozone (UVO-Cleaner, Jelight Company Inc., Irvine, CA, USA) for 90 min prior to measurement. The UV-treated glass (and non-treated glass) was used for additional reference measurements.

### 2.3. Microscopy Analysis of Cells

To understand the effect of ethanol on the morphology and aggregation behavior of the cells, we performed optical and scanning electron microscopy (SEM). Optical microscopy was carried out using a Leica DM750M system (Leica Microsystems GmbH, Wetzlar, Germany) on cells pre-incubated in various ethanol concentrations (*v*/*v*). Specimens were prepared by depositing a droplet of cell suspension onto a microscope slide and placing a clean coverslip over the droplet. Gentle vertical pressure was then applied to obtain a uniform monolayer while minimizing lateral shear. Imaging was performed with the two slides in place to maintain the distribution of cells formed as a function of ethanol concentration on the surface.

SEM imaging was carried out using a JEOL JSM-7800 F instrument (Freising, Germany) similar to the description in reference [38]. Cell suspensions were prepared in various ethanol concentrations and drop-casted on 2 cm by 4 cm glass slides. After drying, the yeast layers were sputter-coated with a ~10 nm film of platinum–palladium (ratio: Pt/Pd = 80/20) using a JEOL JFC-2300HR coater. Scanning electron microscopy was performed at chamber pressures < 1.0 × 10^−4^ Pa with an accelerating voltage of 5 kV, employing magnifications from ~100× to 15,000×. Micrographs were processed and evaluated with built-in JEOL SEM software, version 3.0.

For a detailed comparison of cells between the aged and fresh cells, we used atomic force microscopy (AFM) on an Agilent 5500 (Agilent Technologies, Santa Clara, CA, USA). Measurements employed MSNL-F cantilevers (110–120 kHz, 0.6 N m^−1^ spring constant, nominal tip radius 2–12 nm) operated in intermittent contact (tapping) mode [43]. Images were processed using Gwyddion software, version 2.71 [44].

### 2.4. Cell Metabolic Activity Analysis

Time-resolved metabolic activity was monitored with the resazurin reagent (> 99% purity; Acros Organics, Thermo Fisher Scientific, Geel, Belgium). In metabolically active cells, the blue resazurin is reduced to pink resorufin. An increase in resorufin fluorescence therefore indicates cell population metabolism in real time. A 15 µM solution was prepared in autoclave-sterilized 1× PBS (pH 7.4). Cell suspensions were prepared in 1× PBS and mixed with resazurin at 10% *v*/*v* in six-well plates. Fluorescence from 100 µL aliquots was read on a Tecan Infinite 200 PRO microplate reader (Tecan Trading AG, Männedorf, Switzerland) at 590 nm. Experiments were conducted at a pre-set temperature of 27 °C for ≥4 h in kinetic mode with 5 min sampling. For each condition, a composition-matched blank without cells was recorded. While metabolic rates could be compared visually from the time-dependent plots, we employed a quantitative approach, using the half-time to achieve fluorescence saturation (metabolic half-time, *t_m_*_50_). Rather than manual estimation, the t_m50_ values were obtained by fitting a logistic function in OriginPro (OriginPro, Version 2023b. OriginLab Corporation, Northampton, MA, USA) according to Equation (1).

(1) Y=A2+A1−A21+tt1/2p

where *Y* is the time-dependent fluorescence intensity, *A*_1_ [*Y*(*t* = 0)] and *A*_2_ [*Y*(*t* = ∞)] are constants representing the two limiting values of Y, *p* is the slope, while *t*_1/2_ is the half-time (midpoint between *A*_1_ and *A*_2_), thus representing the half-life of the metabolic activity (*t_m_*_50_) of the cell population [36].

### 2.5. Surface Tension Determination for Ethanol–Water Mixtures

Ethanol–water mixtures ranging from 0% to 99.9% *v*/*v* ethanol were prepared using 99.9% pure ethanol (VWR, Haasrode, Belgium) and deionized Milli-Q water (18.2 MΩ·cm at 25 °C). Surface tension measurements were carried out using a DataPhysics OCA 25 device (DataPhysics, Filderstadt, Germany) equipped with an automated dosing unit and a high-resolution camera (USB 3.0). For each measurement, a pendant drop was produced by continuously dispensing the liquid at 1.00 µL/s until the largest possible undetached drop was achieved. A timing protocol was established to dispense the same volume for each sample. The camera captures the profile of the hanging drop, which is back-lit to enhance edge detection [45]. Recorded drop images were processed in the DataPhysics SCA 22 software module, which detects droplet contour from intensity contrast and fits it with a Young–Laplace model to compute surface tension [46]. The drop shape reflects a balance of forces: surface tension tends to minimize area and round the drop, while gravity pulls it downward and elongates it. Thus, higher surface tension yields a more spherical drop, while lower surface tension produces a more elongated, pear-like profile. Each sample was measured at least five times. In addition to measurements on ethanol–water mixtures, we also analyzed the surface tension of selected Jack Daniel’s, a whiskey with alcoholic content (*v*/*v*) of 40%. Surface tension measurements were performed on volume concentrations of 4, 10, 20, and 40%, with all concentrations prepared by diluting in deionized Milli-Q water.

### 2.6. Cell-Based Htm Monitoring Principle

**Figure 1:**
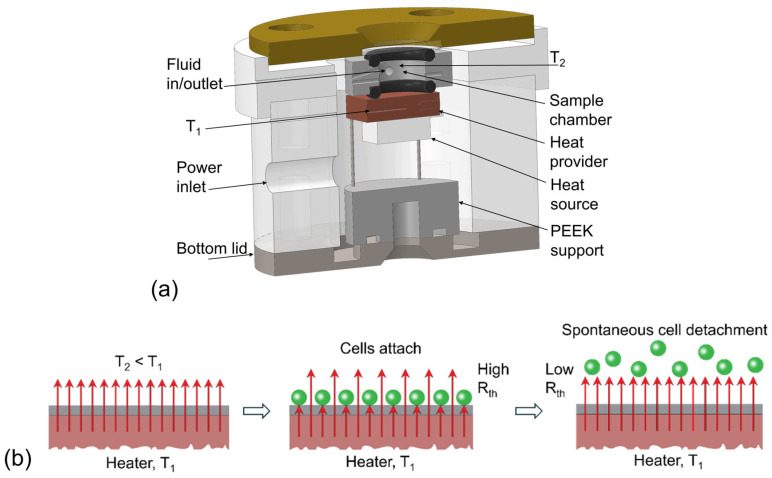
Thermal monitoring of spontaneous detachment. (**a**) Schematic of the heat transfer method (HTM) set-up and spontaneous detachment (**b**). Figure 1a is reprinted from Ref. [36].

The central measurement strategy is based on the recently reported spontaneous cell detachment effect in the presence of a temperature gradient [36]. In summary, when exposed to a fixed temperature gradient, eukaryotic cells, including cancer cells and yeast strains, undergo synchronized, collective, and spontaneous detachment from the solid–liquid interface (chip surface to medium). This detachment occurs within a sharply defined time, *t_d_*_50_, which is sensitive to various physicochemical and metabolic conditions. Here, we exploit the sensitivity of the *t_d_*_50_ parameter to these various conditions. Temperature gradients were generated using the set-up shown schematically in Figure 1a. The set-up, the so-called HTM (heat transfer method) device, is a steady-state technique that measures the heat transfer resistance, *R_th_*, at the solid–liquid interface and has been applied as a transducer platform for various biosensor applications [47]. A heat source of power, *P*, is used to heat a chip from its backside to a constant temperature, *T*_1_, while the temperature of the liquid, *T*_2_, is monitored simultaneously. With regards to cells, cell adhesion attenuates thermal transport, leading to a decrease in *T*_2_. This shows up as an increase in the heat transfer resistance, *R_th_* [*R_th_* = (*T*_1_ − *T*_2_)/*P*], and the opposite holds for cell detachment. Figure 1b illustrates time-dependent spontaneous detachment. The detachment time, *t_d_*_50_, is the time corresponding to the midpoint of the detachment signal, interpreted as the time for half of the cells to detach. In this work, unless stated otherwise, all measurements were performed at a chip temperature of 27 °C. This temperature is close to room temperature and the standard culture temperature (30 °C) used for yeast cells. In addition, by staying at the lower temperature regime, that is, much lower than 37 °C, we avoid the effect of increased convective forces and their associated noise.

## 3. Results and Discussion

### 3.1. Surface Tension of Various Ethanol-Water Mixtures

**Figure 2:**
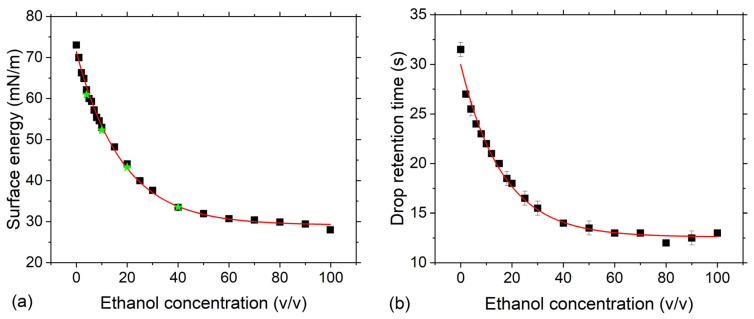
Surface tensions (ST) analysis of ethanol–water and whiskey–water mixtures. (**a**) ST as a function of increasing ethanol volume concentration, showing an exponential decrease from 0% (deionized water, 73.8 ± 0.4 mN/m) to absolute ethanol (99.8% ethanol, 27.8 ± 0.4 mN/m), with Jack Daniel’s volume concentrations of 4, 10, 20, and 40% indicated by the star data points. All measurements were performed at a room temperature of 18.0 ± 0.5 °C. Each data point is an average of at least 5 measurements, and the errors are standard deviations. (**b**) Drop retention time as a function of ethanol concentration.

Surface energy (SE) can have significant effects on cell–material interactions [26,48,49]. Therefore, the surface tension (SE of a liquid) of the ethanol–water mixtures was measured for the entire concentration range from pure water to pure ethanol, enabling observation of the expected decrease in surface tension with increasing ethanol content [50]. As shown in Figure 2a, the surface tension decreases from 73.8 ± 0.4 mN/m in MQ water to 27.8 8 ± 0.4 mN/m in pure ethanol, as expected [50]. The trend is exponential (see fitted line) according to Equation (2).

(2) $$\gamma =\;\gamma_{0}+A\cdot \exp\left(-C/\lambda \right)$$

In Equation (2), *γ*_0_ is the horizontal asymptotic value, *A* denotes the amplitude, *C* is the concentration of ethanol (*v*/*v*), and *λ* is a concentration-scaling parameter (decay constant). The value of *λ* is 19.2 ± 1.1% *v*/*v*, meaning the surface tension decreases by a factor of 0.632 for an ethanol concentration of 19.2%. This data is consistent with results of previous studies. For instance, measurements in reference [50] showed that adding ethanol to water lowers the surface tension from ~72 mNm^−1^ for pure water down to smaller values, e.g., 51.4, 44.8, 37.8, and 33.9 mNm^−1^ at 10, 20, 30, and 40% *v*/*v* ethanol, respectively. For measurements of Jack Daniel’s whiskey, the data points are shown in Figure 2a as green symbols and exhibit the same overall decrease in surface tension, suggesting that ethanol content is the main factor controlling the trend in the diluted whiskey samples. The error bars in Figure 2 are smaller than the symbol size. The very small discrepancy with respect to our data can be attributed to the measurement temperature, as we measured at ambient temperatures as low as 18 ± 0.5 °C. For selected ethanol concentrations, we also analyzed the time it takes before the dispensed drop falls off, or the retention time. The trend in the surface tension data is complemented by the retention time of the drops as a function of ethanol concentration. See Figure 2b with an exponential fit according to Equation (2) (R^2^ > 0.99).

### 3.2. Spontaneous Detachment Dynamics as a Function of Ethanol Concentration

**Figure 3:**
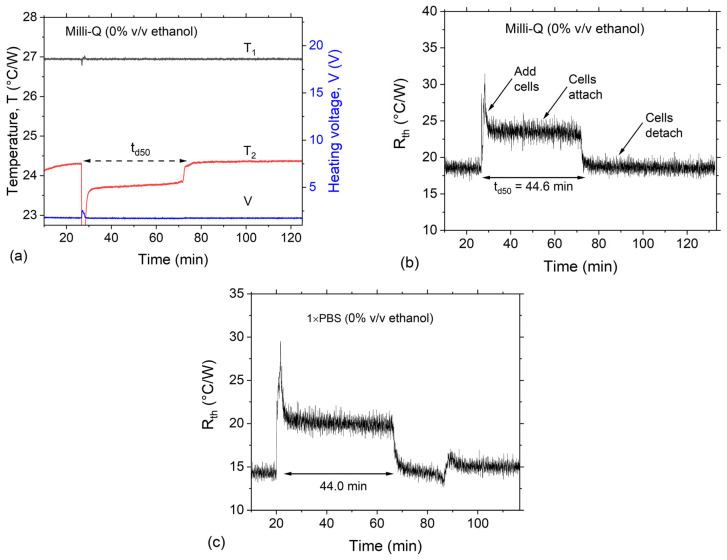
Spontaneous detachment monitoring of yeast in ethanol-free Milli-Q water. (**a**) Typical parameters monitored in real time: chip temperature, T_1_, liquid temperature, T_2_, and heater voltage, V. (**b**) Calculated heat transfer resistance, *R_th_*, from the parameters in (**a**) displaying regimes of cell attachment, cell detachment, and detachment time, *t_d_*_50_. (**c**) Data obtained for cells in 1× PBS showing a similar *t_d_*_50_ value.

Figure 3a shows a typical measurement comparing the liquid temperature, *T*_2_, and the chip temperature, *T*_1_, as a function of time for *S. cerevisiae* cells in ethanol-free medium (MQ water). The chip temperature was preset to 27.0 °C, and measurements were performed at a room temperature of 18.0 ± 0.5 °C on a polyurethane-coated aluminum chip. As shown, following an initial stabilization step in MQ, *T*_2_ decreases slowly to a stable value after the chip surface is exposed to a cell suspension, while *T*_1_ (and heater voltage, *V*) remains constant at the pre-set value. After 44 min, *T*_2_ (and *R_th_*) spontaneously recovers due to collective cell detachment (Figure 3a,b). The detachment time, *t_d_*_50_, was determined by fitting a logistic model as described in ref. [36] and is defined as the time at which the signal recovers to 50% of its total change. Based on six independent measurements (see Figure S1 for other data), the *t_d_*_50_ value is 44.1 ± 2.0 min. As a control, measurements in ethanol-free 1× PBS buffer also produced similar spontaneous detachment times, with an average of 44.0 ± 2.1 min (Figure 3c), suggesting that the effect is less sensitive to changes in osmolarity.

**Figure 4:**
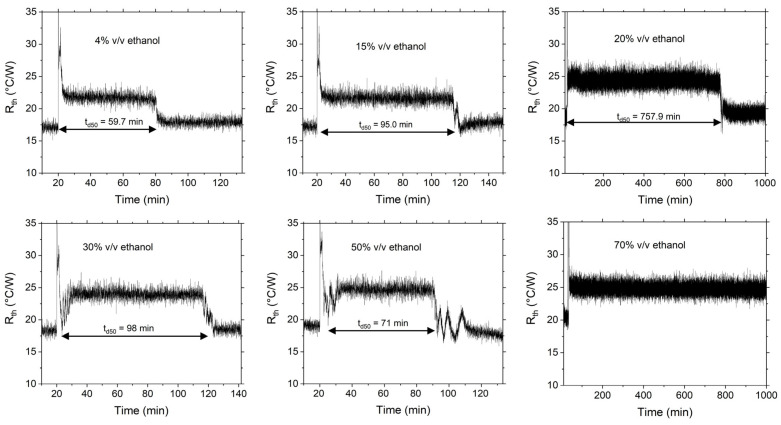
Ethanol-dependent thermal response of yeast cells. Detachment time, *t_d_*_50_, increases with increasing ethanol concentration to a maximum at 20% ethanol. From 20% ethanol, the detachment time decreases. No detachment is measured for 70% *v*/*v* ethanol.

Using the same measurement protocol as in Figure 3, yeast suspensions preincubated in various ethanol concentrations (*v*/*v* %) for 1 h were measured. Overall, data was acquired for 0, 2, 4, 6, 10, 15, 20, 25, 27.5, 30, 40, 50, 60, and 70% ethanol. Figure 4 compares the raw *R_th_* data for time-dependent cell attachment and detachment behaviors for representative measurements of 0, 15, 20, 30, 50, and 70%. See Figures S1–S6 for all other data. Overall, spontaneous cell detachment occurs for ethanol concentrations up to 50%. Specifically, between 0 and 20%, the detachment time increases: 44.6 min in 0%, 95.0 min in 15% and 758 min in 20%. The long detachment time for 20% ethanol was unexpected but very persistent and reproducible, as seen in the multiple measurements presented in Figure S5. Interestingly, the change in detachment time from 15% to 20% is very sharp. Beyond 20%, the detachment time decreases. No detachment occurs in 60 and 70%; thus, at these ethanol levels, the cells are dead, in addition to generally clustering out of the suspension and onto the chip, as explained in Section 3.3.

**Figure 5:**
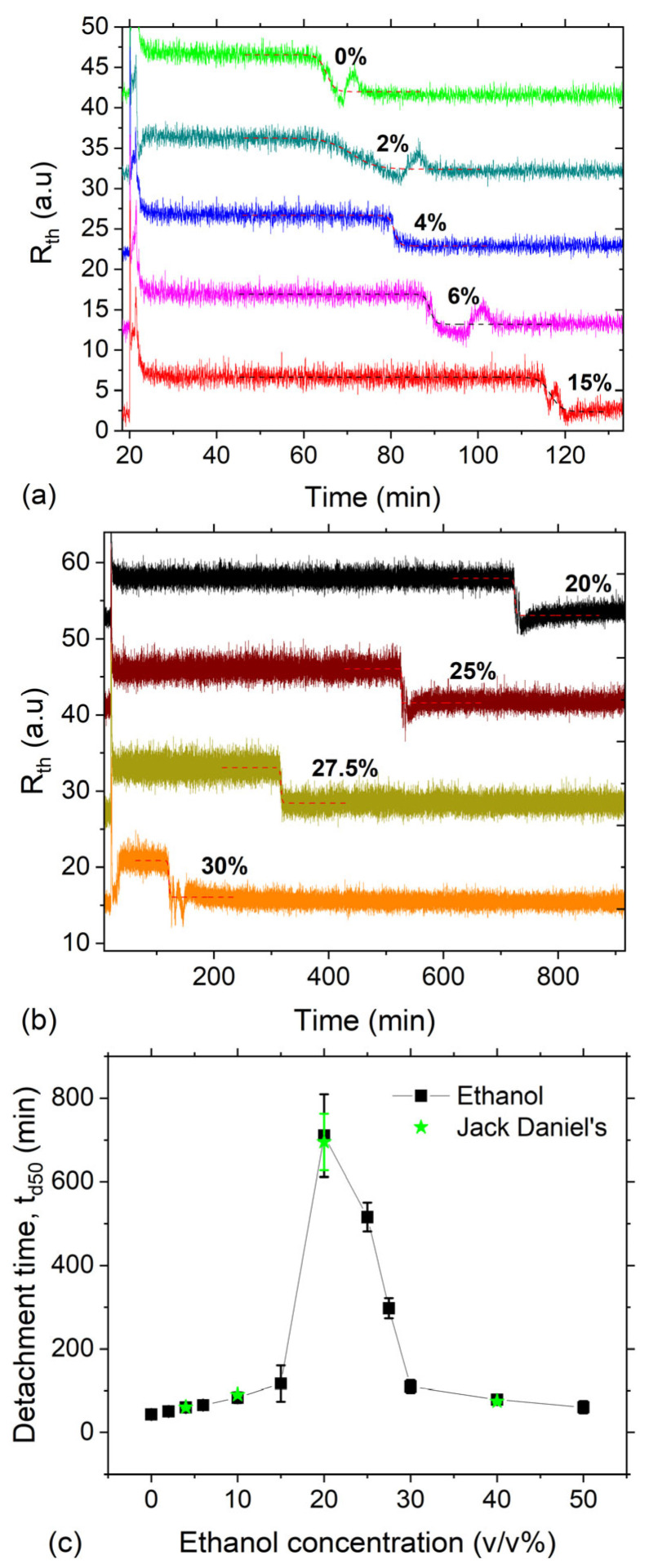
Ethanol-dependent yeast detachment. (**a**,**b**) Time-dependent *R_th_* data of representative ethanol concentrations showing an increase in detachment time up to 20% and a decrease for higher ethanol concentrations. (**c**) Plot of cell detachment time as a function of ethanol concentration for at least three measurements for each data point. The error bars are standard deviations. Detachment time increases gradually up to 15% ethanol before a sharp increase to a maximum at 20% and a final decreasing trend until 50%. Data points for Jack Daniel’s whiskey (green stars) for the undiluted 40% alcohol and dilutions in Milli-Q water at 4, 10, and 20% alcohol (*v*/*v*) consistent with the ethanol trend. All data measured in triplicate; error bars (often smaller than the symbol size) represent standard deviation.

For easy visualization, Figure 5a compares the *R_th_* response for various ethanol concentrations between 0 and 15% in one panel, highlighting the increasing trend in detachment time, while Figure 5b depicts the decreasing trend in detachment time for yeast in 20% and higher concentrations up to 30%. Data in Figure 5a,b are displaced along the vertical axis for easy visual comparison, and the dottle lines are examples of the logistic fits used to determine the detachment half-time, *t_d_*_50_.

Figure 5c displays the full range of data and all ethanol concentrations measured, except for 60 and 70%, for which no cell detachment occurred (Figure S6). All data points are an average of at least three independent measurements, and the errors are standard deviations.

To demonstrate the applicability of the method in a chemically complex and practical sample matrix, we additionally tested a commercial alcoholic beverage, Jack Daniel’s whisky, as well as diluted samples. Its 40% *v*/*v* alcohol content enabled validation over a substantial portion of the ethanol calibration range shown in Figure 5c, whereas lower alcohol matrices, such as wine or beer, would access only a narrower concentration window. The undiluted whiskey (40%) and the diluted samples, corresponding to approximately 4, 10, and 20% ethanol, produced responses consistent with those obtained from the ethanol calibration measurements. The data is shown as stars in Figure 5c. Each data point is an average of at least three independent measurements, and the errors are standard deviations. These results indicate that the yeast-based sensing approach is not limited to simple ethanol-in-water solutions but can also be applied to real alcoholic beverage samples. The agreement between the surface tension data obtained for the ethanol–water mixtures and the whiskey samples indicates that the sensor response in the whiskey matrix is primarily attributable to ethanol. Importantly, the present method is proposed as a proof-of-concept analytical approach rather than as a ready-to-use device for all applications. Fermentation monitoring represents one possible future application, in which the method could be used to test samples extracted or obtained during the fermentation process. As demonstrated by the whisky measurements, the method may also be useful for ethanol quantification in beverages such as beer, including the distinction between alcoholic and non-alcoholic beers, as well as wine, spirits, and related products.

It is also worth noting that in addition to following the general trend, cells exposed to the 20% whisky sample displayed the uniquely long detachment times observed for 20% ethanol. This confirms that the effect is associated with ethanol concentrations around this level.

**Figure 6:**
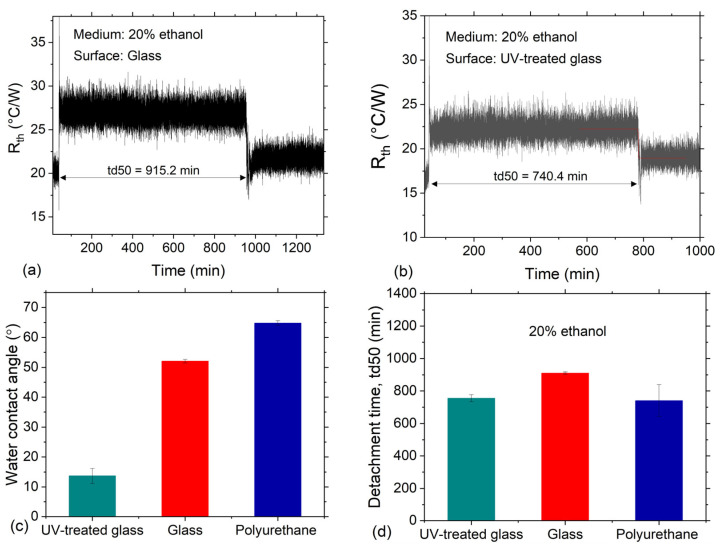
Evaluation of surface-dependent cell detachment of yeast cells in 20% *v*/*v* ethanol. Detachment behavior on glass (**a**) and highly hydrophilic UV-treated glass (**b**), showing long detachment times, similar to the values on PU-coated Al. (**c**,**d**) Comparison of contact angles (**c**) and detachment times (**d**) for all three materials used, showing long detachment times > 600 min for all surfaces and no direct correlation with surface hydrophobicity.

It is well-known that surface wetness (and surface energy) significantly affects the adhesion of cells to surfaces [26,49]. However, measurements of spontaneous cell detachment as a function of surface chemistry on polyurethane, gold, and glass (further differentiated by UV-exposed and silanized) showed that *t_d_*_50_ was independent of the surface [36]. To understand whether the long detachment time for cells in 20% *v*/*v* ethanol also occurs on other surfaces, we performed similar measurements on materials with different surface hydrophobicity, evaluated through water contact angle (CA) measurements, including UV-treated glass (CA 13.7 ± 2.5°) and glass (CA 52 ± 0.6°). Polyurethane, on which all other measurements were obtained, has a contact angle of 64.8 ± 0.8°. As shown in Figure 6, data on glass (Figure 6a) and UV-treated glass (Figure 6b) also display long detachment times of 915.2 min and 740.4 min, respectively; in each case, it is more than 10 times the value in the absence of ethanol [36]. Therefore, the long detachment time observed in the presence of 20% ethanol appears to be a general phenomenon across the surfaces analyzed. Notably, the trend in surface hydrophobicity (Figure 6c) did not correspond to the variation in detachment time: the value on glass was markedly longer, whereas values on UV-treated glass and polyurethane were comparable within experimental error (Figure 6d).

From the perspective of interfacial energies, an important factor that affects both cell adhesion and cell membrane interactions is liquid surface tension. The surface tension plays a role in interfacial energies at cell–liquid and solid–liquid interfaces and consequently determines the nature of the interactions at the chip–cell interface [51]. This relationship, based on the thermodynamic theory of cell adhesion, is driven by the tendency to minimize the total interfacial energy [52]. This means that cells preferentially adhere to surfaces offering the lowest energy [26,51]. From the perspective of the DLVO theory, ethanol can affect cell adhesion in various ways: (1) increasing ethanol decreases the dielectric constant of the mixture, which in turn weakens the electrostatic repulsion (lower barrier) and increases van der Waals forces; (2) ethanol weakens the degree of ionization of surface groups on both cells and substrates, lowering surface charge and decreasing electrostatic repulsion, further lowering the potential barrier; and (3) ethanol lowers surface tension, as seen in Figure 1, which reduces hydration forces. This has the effect of decreasing the very strong short-range repulsive forces, thus enhancing adhesion and preventing cell detachment [53]. With regards to the general detachment trend displayed in Figure 4, Figure 1 shows a continuous decrease in the surface tension as a function of ethanol concentration, with no observable anomalies at or around 20% ethanol. However, fitting the data with the exponential function of Equation (2) gives a decay constant that matches the detachment time in ~20% ethanol, meaning that the main changes in the surface tension as a function of ethanol occur in the 0–20% range and slow down afterwards, although this does not offer a direct explanation with regards to the delayed detachment. A well-known property of ethanol–water mixtures that varies with ethanol concentration is viscosity. However, the maximum viscosity occurs at approximately 50% ethanol. We additionally verified this trend using QCM-D measurements. The data confirms the trends reported in the literature; thus, the observed detachment behavior is unlikely to be primarily driven by viscosity changes in the ethanol–water medium in which the cells are suspended.

While this work offers a method for cell-based ethanol detection, it may also provide insight into the mechanisms of ethanol tolerance of yeast cells, that is, from the perspective of biomechanical changes. Specifically, the dramatic delay in cell detachment for cells in 20% *v*/*v* ethanol may indicate the existence of a mechanism that peaks at this level of ethanol or the existence of competing mechanisms, with a transitional point at 20%. Many mechanisms are linked to the ethanol tolerance of yeast, ranging from genetic modulations (altering posttranscriptional levels of gene expression) [54,55,56] to changes in cell membrane fluidity [57]. Studies show that the specific response mechanism depends on the level of ethanol itself. For instance, as reported in reference [57], comparative transcriptome profiling of the two yeast strains (*S. cerevisiae* (BR20) and *S. cerevisiae* (F23) under different ethanol concentrations revealed distinct adaptive strategies. When cells were exposed to 10% *v*/*v* ethanol, pathways linked to amino acid metabolism were most closely associated with improved stress tolerance. In contrast, under the more severe condition of 18 vol% ethanol, fatty acid metabolism emerged as the primary contributor to survival. These findings highlight that the mechanisms underlying ethanol resistance shift depending on stress level, supporting earlier studies that showed that separate sets of genes are engaged under mild versus severe ethanol stress [58].

Membrane fluidity/membrane integrity plays a key role in yeast ethanol tolerance [55,57,59,60]. The way fatty acids are packed within the membrane directly influences its fluidity, which is essential for preserving membrane stability/integrity and can be strongly influenced by ethanol [59]. However, there is debate in the literature, with some studies indicating that higher membrane fluidity improves ethanol tolerance, while others suggest the opposite [57]. This variation may come from differences in yeast strains and the methods used to measure fluidity. These methods mainly utilize strategies based on fluorescent probes to evaluate membrane fluidity. These include, for instance, electron spin resonance (ESR), AFM force spectroscopy, NMR spectroscopy, fluorescence recovery after photobleaching (FRAP), fluorescence correlation spectroscopy (FCS), and single particle tracking (SPT). A few studies show that ethanol levels around 20% cause a sharp disruption in yeast membranes. For instance, in their study, Learmonth and coworkers assessed yeast membrane dynamics using both DPH anisotropy and Laurdan GP and showed that when *S. cerevisiae* was exposed to 20% ethanol, they observed a sharp rise in membrane fluidity, which was later followed by partial or inconsistent stabilization [61]. These results are similar to Yang et al., who showed a significantly higher loss of membrane integrity for yeast cells (*S. cerevisiae* 288C) when exposed to 18–20% ethanol [57]. All of these studies were, however, carried out in the presence of cell culture medium in which ethanol levels are prone to changes due to fermentation of the medium, resulting in unaccounted in situ changes in the ethanol concentration under study. Unlike studies in the literature, our study was performed in the absence of culture medium, thus guaranteeing observation of the intrinsic yeast response at the macroscopic level to a constant ethanol concentration.

Overall, the literature indicates that the pronounced peak in detachment behavior near ~20% *v*/*v* ethanol is best explained by hard biophysical limits, such as changes in membrane fluidity and integrity, altered interfacial energetics, and solvent-induced protein destabilization, rather than a discrete, programmed cellular threshold. These interactions, acting together or in part, may be responsible for promoting higher cell density and thus stronger cell–cell binding while also increasing the interaction area at the surface and thereby enhancing cell–chip binding.

With regards to applying the calibration curve for ethanol quantification, the 0–20% *v*/*v* region provides the primary quantitative working range for direct single-shot measurements, which is particularly relevant for fermentation monitoring where ethanol typically evolves within this regime. The limit of detection (LOD) for ethanol based on the *t_d_*_50_ data was estimated to be 1.3% ethanol. This value was obtained from an exponential fit of the calibration data over the 0–20% ethanol concentration range (R^2^ = 0.97). The detection threshold was defined as the blank response plus three times its standard deviation, corresponding to *t_d_*_50_ = 43.1 min + 3 × 2.0 min = 49.1 min. Interpolation of this threshold value on the fitted calibration curve yielded an LOD of 1.3% ethanol. To ensure unambiguous quantification above 20%, we can employ a fixed 1:1 (50%) dilution protocol and record detachment times for both the undiluted sample and its diluted aliquot. The paired measurements then yield a unique concentration estimate because the diluted aliquot falls within the calibrated monotonic range, enabling robust back-calculation using the known dilution factor. In addition, with regards to the assay time, quantification of the 20% concentration does not require monitoring until the final detachment signal. Because detachment time is concentration-dependent, exceeding specific temporal thresholds can confirm concentration levels.

Thus far, this work has focused on ethanol detection in ethanol–water mixtures, with practical applicability demonstrated through measurements in a representative spirit sample, Jack Daniel’s whiskey, used as a complex matrix. The agreement between the surface tension trends observed for ethanol–water mixtures and whiskey samples is consistent with ethanol being the dominant contributor to the sensor response. However, further optimization of the method is required, for instance, by assessing sensor performance in the presence of other compounds that commonly coexist with, or contaminate, ethanol-containing solutions. It should be noted that all data discussed here were measured at a chip temperature of 27 °C, as described in the Materials and Methods section. Measurements performed at different temperatures would alter the corresponding *t_d_*_50_ values [36].

### 3.3. Morphological Analysis

The morphology of cells as a function of ethanol concentration was assessed using SEM and optical microscopy. For SEM, cells were prepared for imaging by dry casting, as described in the Materials and Methods. Equal volumes of cell suspensions were deposited onto glass slides, and the liquid was allowed to evaporate. Therefore, regardless of the ethanol concentration, the same number of cells was deposited on the slides. Figure S8 presents SEM micrographs of yeast cells exposed to ethanol concentrations ranging from 0 to 70% (*v*/*v*) in deionized water. At low ethanol levels (0–20%), cells exhibited an elongated morphology and tended to adhere to each other along their long axis. This elongated morphology was no longer apparent at ethanol concentrations ≥ 30%, where cells appeared uniformly spherical. The observed transition in morphology suggests that ethanol influences the physical integrity of yeast cells: at ≤20% ethanol and below, membrane integrity appears more strongly affected, consistent with reports in references [57,60,61], while at ≥30%, cells maintain their overall spherical form. The preservation of cell shape at higher ethanol levels is consistent with a fixation-like effect [62].

For optical imaging (Figure S9), samples were prepared by placing a drop of cell suspension between two glass slides to create a monolayer of cells. Optical micrographs revealed pronounced clustering of cells into distinct patches at 70% ethanol, whereas lower ethanol concentrations produced a more dispersed distribution. This clustering likely accounts for the absence of cell detachment observed at 60% and 70% ethanol, in addition to other well-known effects, such as protein denaturation. The clustering behavior is further investigated in Section 3.4.

### 3.4. Time-Dependent Od600 Analysis of Cells

**Figure 7:**
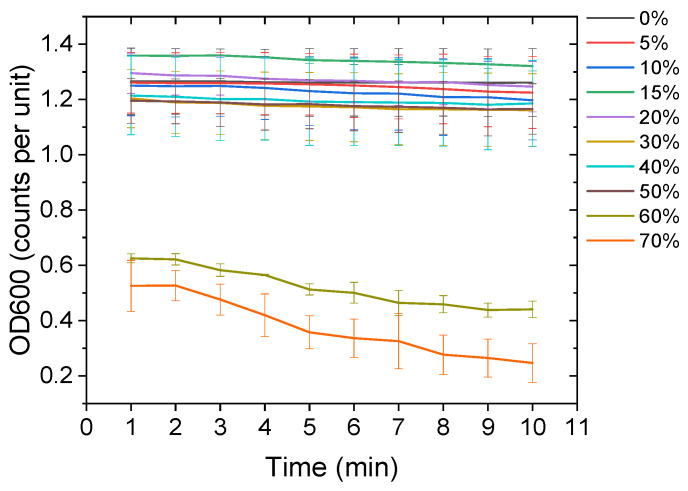
OD600 values for 60 and 70% ethanol depicting a much lower and decreasing trend as a function of time compared to yeast in lower ethanol concentrations from 0 to 50%. The effect is more pronounced for 70% ethanol.

In addition to the cell metabolic activity effects, we hypothesized that the lack of detachment for 60 and 70% ethanol can be attributed to the fact that cells quickly cluster to avoid ethanol, an effect that is observed with the naked eye when a yeast suspension is prepared in 70% ethanol. To elucidate this, OD600 measurements were performed as a function of time. Cells sediment with time; thus, measuring the OD600 as a function of time gives direct information on the sedimentation rate of the cells for different ethanol concentrations and provides insights into the clustering behavior of the cells, while initial OD600 values give information on the cell count resulting from ethanol treatment. As shown in Figure 7, the OD600 values remain moderately constant with 10 min of measurement for ethanol concentrations ranging from 0 to 50% *v*/*v*, which also corresponds to the concentration range that displays detachment under a temperature gradient.

There is no special behavior for the OD600 values at 20% *v*/*v* ethanol, which means that short-term colloidal demixing is not responsible for the peak *t_d_*_50_ value. On the contrary, the data at 60 and 70% *v*/*v* ethanol display almost instantaneous decrease in OD600 values, as seen from the 1st minute. The cell concentrations were the same for all ethanol concentrations; thus, the low values are due to fast sedimentation within the first minute. The OD600 values also continue to decrease over the 10 min measurement time compared to the values for 50% ethanol and lower. Therefore, one can hypothesize that at 60 and 70% *v*/*v* ethanol, the solvent properties change so drastically (dielectric constant, hydration layer disruption, protein denaturation at the cell wall, etc.) that the suspension loses stability, resulting in colloidal demixing, where yeast cells quickly separate out of the ethanol phase, aggregate, and settle at the bottom. These results are supported by the microscopy data in Figure S9a, which shows aggregation of cells in 70% ethanol compared to cells in 0, 10, 20, and 40% ethanol.

### 3.5. Effect of Cell Metabolic Activity—Towards a Cell Quality Monitoring Assay

**Figure 8:**
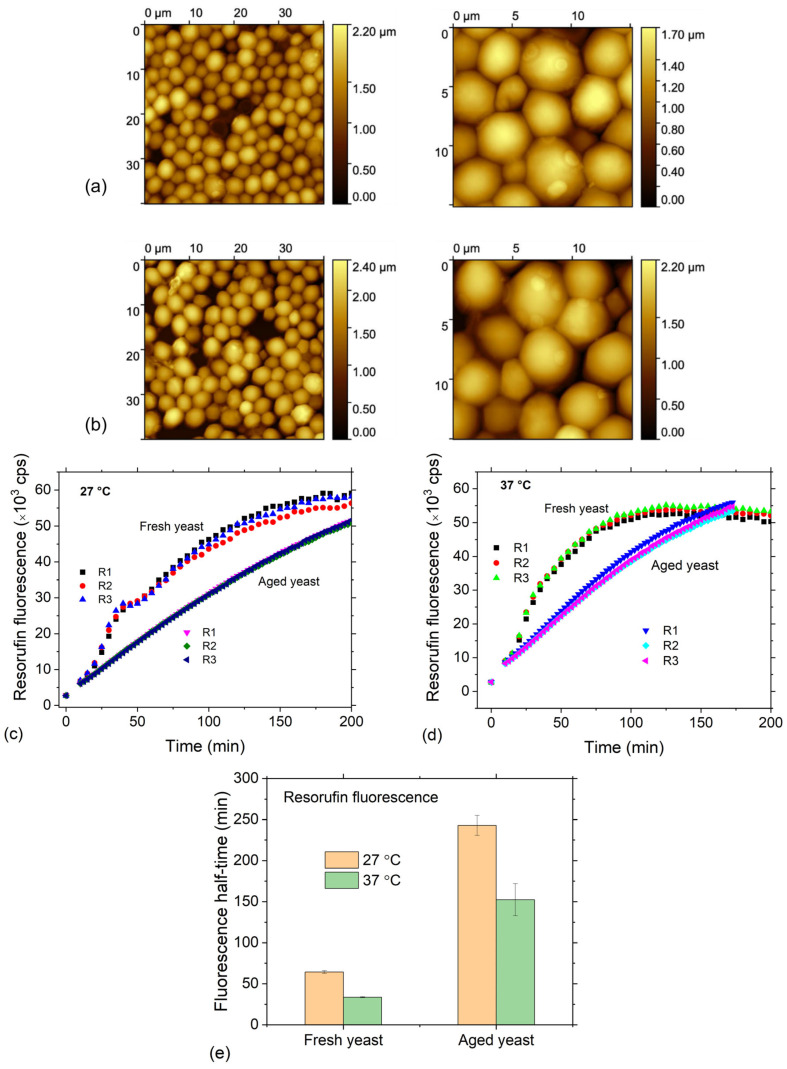
Morphological and metabolic characterization of yeast cells. (**a**,**b**) AFM images of yeast cells comparing aged (**a**) and fresh (**b**) cells. Images of both cell types show similar features typical of budding yeast cells. (**c**,**d**) Comparison of yeast metabolic activity between aged and fresh yeast cells for two temperatures, 27 °C (**c**) and 37 °C (**d**), showing faster and earlier saturation for the fresh cells. (**e**) Resorufin fluorescence half-time, quantifying metabolic activity half-time, *t_m_*_50_ between aged and fresh yeast cells at 27 °C and 37 °C. The error bars in (**e**) are the standard deviations. In (**c**,**d**), R1, R2, and R3 are repeated measurements under the same conditions.

Considering the importance of cell vitality in various cell-based applications [63], the objective here was to evaluate the correlation between yeast cell quality and spontaneous detachment kinetics and how this is, in turn, modulated by ethanol concentration. The upper panels in Figure 8 compare the AFM images of aged (a) and fresh (b) *S. cerevisiae*. In Figure 8a,b, the right panels are magnified views of the samples shown on the left. As shown, fine details of the cell attributes are visible, such as emerging buds, from both the aged and fresh cells. However, there are no discernible differences in the shape and size distribution of the cells, meaning that the physical integrity of the aged cells seems to be preserved. To assess the metabolic activity of aged and fresh cells, the resazurin test was used [64,65,66]. Figure 8c,d compare the fluorescence of resorufin as a function of time between fresh and aged cells for two temperatures, 27 °C and 37 °C, respectively, over three repeated measurements each (R1, R2, R3). As expected, the rate of fluorescence increase is faster, with a shorter saturation time for fresh yeast compared to aged yeast. Figure 8e compares the resorufin fluorescence half-lives between aged and fresh cells determined as described in Equation (1) for two temperatures, 27 °C and 37 °C. As depicted, half-time is shorter for the fresh cells regardless of the measurement temperature. This confirms that these cells indeed display high metabolic activity. It should be noted that the exact fraction of colony-forming cells (CFU) in the fresh and aged yeast populations was not determined in this study. Accordingly, the fresh and aged yeast populations are compared here only in terms of their relative metabolic activity and cellular vitality, rather than as quantitatively defined fractions of viable cells.

**Figure 9:**
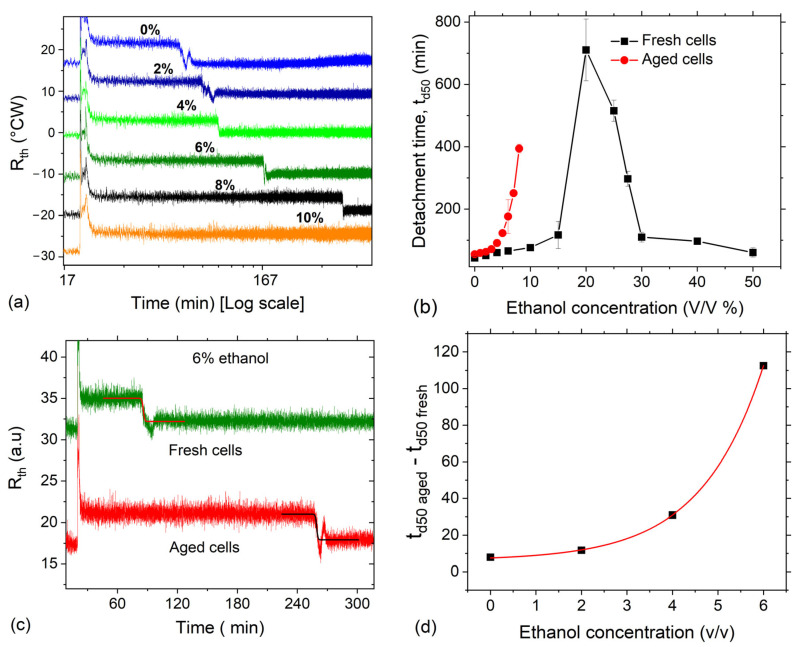
Assessing cell quality through spontaneous detachment. (**a**) Combined plots comparing the detachment response of aged yeast cells, with data displaced along the vertical axis for easy visualization. No detachment is observed for cells in 9% and higher ethanol concentrations. (**b**) Detachment time as a function of ethanol concentrations showing an exponential trend up to 8% *v*/*v* ethanol for aged cells and 50% *v*/*v* ethanol for fresh cells. (**c**) Raw *R_th_* data comparing the spontaneous detachment response between fresh and aged yeast for 6% ethanol. (**d**) Difference between *t_d_*_50_ of aged and fresh yeast cells for selected ethanol concentrations, 0, 2, 4, and 6%, displaying an exponential profile (R^2^ = 0.9999). The data points in (**b**) and, consequently, (**d**) are averages from at least three measurements, and errors are standard deviations.

Figure 9a displays the time-dependent *R_th_* response from measurements on aged yeast cells in various ethanol–water mixtures. As already mentioned, even though these cells are aged, they still show a level of biological activity, as illustrated in the resazurin test in Figure 8c–e. The raw *R_th_* data for the other ethanol concentrations are shown in Figure S10.

Like the fresh cells, these cells also display spontaneous detachment behavior, with a detachment time that increases with increasing ethanol concentration. The cells are only responsive for ethanol concentrations up to 8%, above which no detachment is measured. This behavior is markedly different from fresh cells, which show detachment for ethanol concentrations up to 50% *v*/*v*. Figure 9b (curve with circular symbols) shows a comparison of the average detachment time, *t_d_*_50_, of the aged cells as a function of ethanol concentration. Figure 9b also compares the *t_d_*_50_ values between aged and fresh cells across the full range of ethanol concentrations over which detachment occurs, while Figure 9c shows, as an example, the raw *R_th_* data at 6% *v*/*v* ethanol for the two cells.

One can identify three distinct features when comparing the response of the two cell categories. (1) In ethanol-free medium (0% ethanol), the difference between the *t_d_*_50_ values is small. That is, for aged cells, *t_d_*_50_ is 54.8 ± 2.5 min, only 10 min longer than the detachment time for the fresh cells of 44.1 ± 2.0 min. As ethanol concentration increases, the response (ability to detach) of the aged cells dramatically decreases, while fresh cells continue to respond (Figure 9b). For aged cells, ethanol levels as low as 9% completely suppress detachment, while fresh cells tolerate detachment at much higher levels. This highlights that the response of cells to ethanol is strongly dependent on the metabolic activity (or vitality) state of the cells. The difference in response time (*t_d_*_50_) values is displayed in Figure 9c, showing an exponential dependence (Equation (2)).

**Figure 10:**
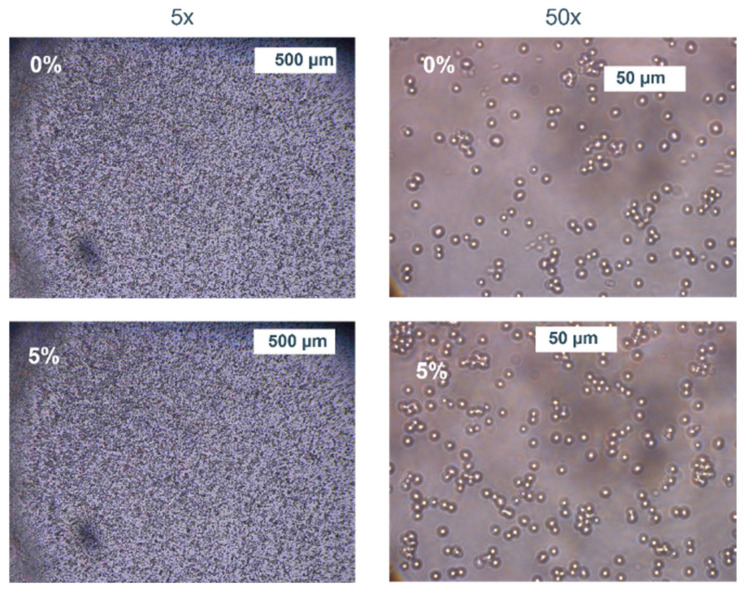

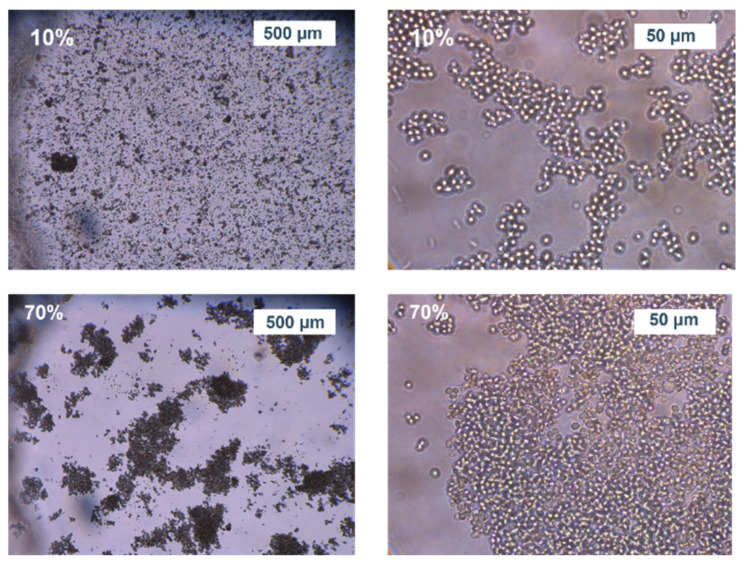
Optical micrographs of aged yeast cells in various ethanol concentrations on glass slides. Representative micrographs showing dispersion/aggregation behavior of aged yeast cells on glass for various ethanol concentrations. The first and second columns correspond to images acquired with 5× and 50× microscope objectives. Cells at 10% ethanol show significant aggregation, which is even higher for 70% ethanol.

Figure 10 presents representative optical micrographs of cells on glass surfaces prepared as described in the Materials and Methods. The left panels show images acquired at 5× magnification, providing an overview of cell coverage and spatial distribution across the surface, whereas the right panels show images acquired at 50× magnification, allowing cellular distribution and local aggregation patterns to be assessed at higher spatial resolution. In the absence of ethanol, the cells formed a relatively homogeneous and dispersed layer, and a similar distribution was observed in the presence of 5% ethanol, but with moderate aggregation. In contrast, pronounced cell agglomeration became apparent at 10% ethanol and was most pronounced at 70% ethanol. A similar agglomerated distribution was also observed at intermediate ethanol concentrations above 10%. This also corresponds to the ethanol range for which detachment does not occur. Overall, this data highlights the utility of spontaneous detachment monitoring under a temperature gradient as a sensitive method for not only quantifying the levels of ethanol but also for evaluating the metabolic activity of the cells based on detachment kinetics as well as ethanol levels to which the cells can still respond.

While the sensor platform demonstrates clear potential for cell quality assessment, this does not compromise its application for ethanol detection. Instead, the dependence of detachment time on cell vitality can be viewed as an advantage, as it enables in situ quality control of the cells to be used. Thus, prior to quantitative ethanol sensing, a reference measurement at a defined ethanol concentration can be used to verify that the yeast cell population exhibits the expected detachment response.

### 3.6. Comparison with Established Ethanol Detection Methods

Compared with alcohol dehydrogenase (ADH)- and alcohol oxidase (AOX)-based biosensors and commercial hand-held alcohol meters, the present method is slower but considerably simpler. Enzyme-based biosensors can provide seconds-scale responses but usually require purified immobilized enzymes, modified electrodes, or films, supporting reagents such as NAD^+^ (nicotinamide adenine dinucleotide) and dedicated electrochemical readout [1]. Similarly, commercial alcohol meters are rapid but typically rely on specialized refractive index or density measurement hardware and may require matrix- or beverage-specific calibration [6]. In contrast, the present platform uses only a heater, two temperature sensors, and yeast as a whole-cell reporter, supporting low-cost operation with minimal reagents and consumables [48]. The use of *S. cerevisiae* also offers a potential sustainability advantage, particularly in fermentation-related contexts where yeast is already present and may be valorized as a process-associated sensing material. In addition, the platform enables monitoring of reporter cell quality, providing a functional distinction from conventional physical-property-based alcohol meters. Because spontaneous cell detachment dynamics have previously been shown to depend on temperature under different physicochemical conditions [36], the assay time may also be further reduced by tuning the operating conditions while preserving reporter cell integrity and signal quality.

## 4. Conclusions

We demonstrate that spontaneous detachment of yeast cells under a temperature gradient provides a robust, label-free readout for ethanol quantification. Remarkably, the detachment time exhibits a distinctive, non-monotonic dependence on ethanol concentration with a sharp maximum at 20% *v*/*v* that is insensitive to substrate hydrophobicity. At higher concentrations (≥60% *v*/*v*), cells fail to detach. The pronounced maximum at 20% *v*/*v* was unanticipated and might reflect a mechanism-based transition in which membrane integrity is maximally compromised, enhancing cell–cell aggregation and cell–surface contact; at higher ethanol levels, alternative mechanisms likely dominate, such as protein denaturation. Elucidating the physico-chemical and biological origins of this transition might be essential to define its generality across strains and conditions as well as to further highlight its applications.

From an analytical perspective, this calibration behavior enables direct ethanol quantification within the monotonic 0–20% *v*/*v* regime, which is relevant, for example, for fermentation monitoring. Concentrations above 20% *v*/*v* can be quantified unambiguously by measuring a fixed (e.g., 1:1) dilution in parallel with the undiluted sample to shift the response into this calibrated window, after which the original concentration is recovered through straightforward back-calculation using the known correction factor. As a step towards practical application, measurements performed with Jack Daniel’s whiskey demonstrate that the responses obtained at selected alcohol concentrations agree with the ethanol calibration curve, thereby validating the method for ethanol quantification in a complex matrix.

We further show that the proposed cell-based biosensor couples ethanol sensing with cell quality assessment. Yeast displaying high metabolic activity responds to up to ~50% *v*/*v* ethanol, whereas metabolically less active cells lose detachment response above ~8%. Complementary analysis using resazurin assay, microscopy (AFM/optical), and OD600 indicate that this contrast primarily reflects vitality rather than morphology, population heterogeneity, or cell count, which appear comparable between aged and fresh cells. The dual functionality, that is, quantitative ethanol detection paired with vitality screening, addresses practical needs in beverage quality control, biofuel process supervision, and rapid screening where robustness under ethanol stress is critical.

Overall, this label-free method operates on unmodified whole cells, requires no chemical reagents, and needs only simple heating and temperature monitoring, and any chip material can be used. With minimal sample preparation and compatibility with real-time measurements, it delivers a low-cost assay for ethanol quantification, vitality screening, and broader studies of cellular stress.

## Data Availability

The data is contained within the article and Supporting Information. Data in alternative formats is available from the corresponding author upon request.

## Supplementary Materials

The following supporting information can be downloaded at: https://www.mdpi.com/article/10.3390/bios16070355/s1, Figure S1: Spontaneous yeast response to 0% ethanol. Figure S2: Spontaneous yeast response to 2, 4 and 6% ethanol. Figure S3: Yeast response to 10% ethanol. Figure S4: Spontaneous yeast response to 15% ethanol. Figure S5: Spontaneous yeast response to 20% ethanol. Figure S6: Spontaneous yeast response to 25–70% ethanol. Figure S7: Additional measurements showing the spontaneous yeast response to 20% ethanol on glass and uv-treated glass. Figure S8: SEM analysis of yeast as a function of ethanol concentration. Figure S9: Optical microscopy and dynamic OD600 analysis of cells compared by ethanol concentration. Figure S10: Influence of ethanol on the spontaneous detachment of aged yeast cells.
