# Supplementary material for: A Label-Free Cell-Based Biosensor Method for Ethanol Quantification Using Temperature-Induced Spontaneous Cell Detachment
Source: Biosensors (Basel). 2026 Jun 25;16(7):355. doi: 10.3390/bios16070355 (PMC13406514; doi:10.3390/bios16070355)
Supplement: Supplementary file 1 [file biosensors-16-00355-s001.zip › biosensors-4306216-supplementary.pdf]

Supporting Information

A label-free cell-based biosensor method for ethanol quantification using temperature-induced spontaneous cell detachment

Derick Yongabi[1], Alex Krane[1], Heloisa E.G. Ramos[2], Sofia X. Bustia[2], Jonas Gruber[2], Michael J. Schöning[3], Frank Delvigne[4], Patrick Wagner[1]

[1] KU Leuven, Department of Physics and Astronomy, Laboratory for Soft Matter and Biophysics, Celestijnenlaan 200 D, B-3001 Leuven, Belgium.

[2] Universidade de São Paulo, Instituto de Química, Av. Prof. Lineu Prestes, 748, CEP 05508-000, São Paulo, SP, Brazil.

[3] Aachen University of Applied Sciences, Institute of Nano- and Biotechnologies (INB), Heinrich-Mußmann-Straße1, D-52428 Jülich, Germany.

[4] University of Liège, Gembloux Agro-Bio Tech, Terra Research and Teaching Centre, Microbial Processes and Interactions (MiPI), Av. de la Faculté d'Agronomie 41/13, 5030 Gembloux, Belgium.

* Corresponding author:

Dr. Derick Yongabi
KU Leuven, Department of Physics and Astronomy,
Laboratory of Soft Matter Physics and Biophysics ZMB,
Celestijnenlaan 200 D, B-3001 Leuven, Belgium
E-mail: Derick.Yongabi@kuleuven.be

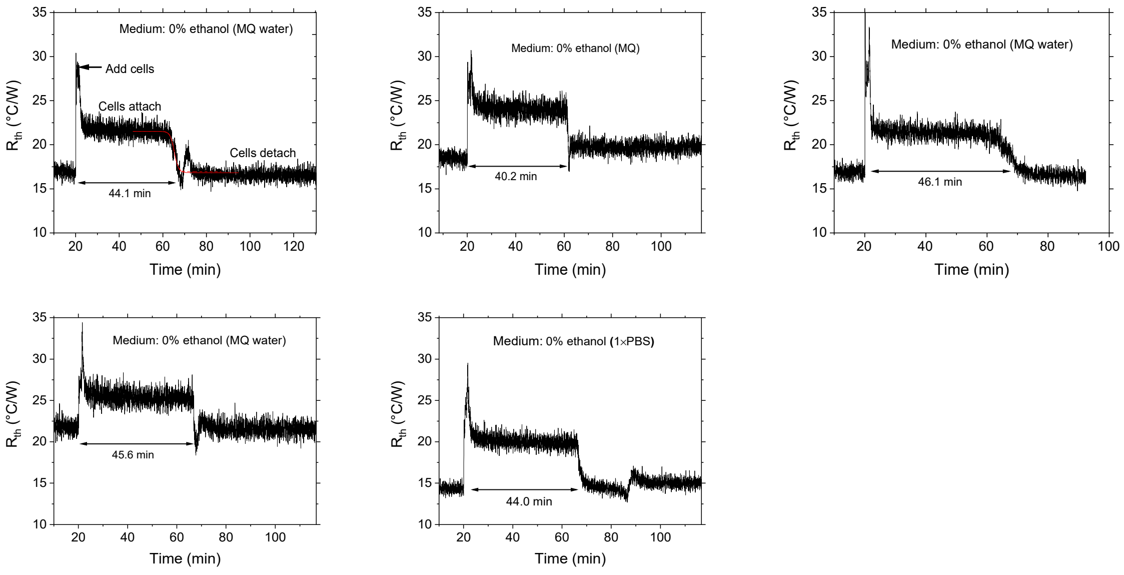

***Figure S1**: Spontaneous yeast response to 0% ethanol. All measurements were performed at 27°C.*

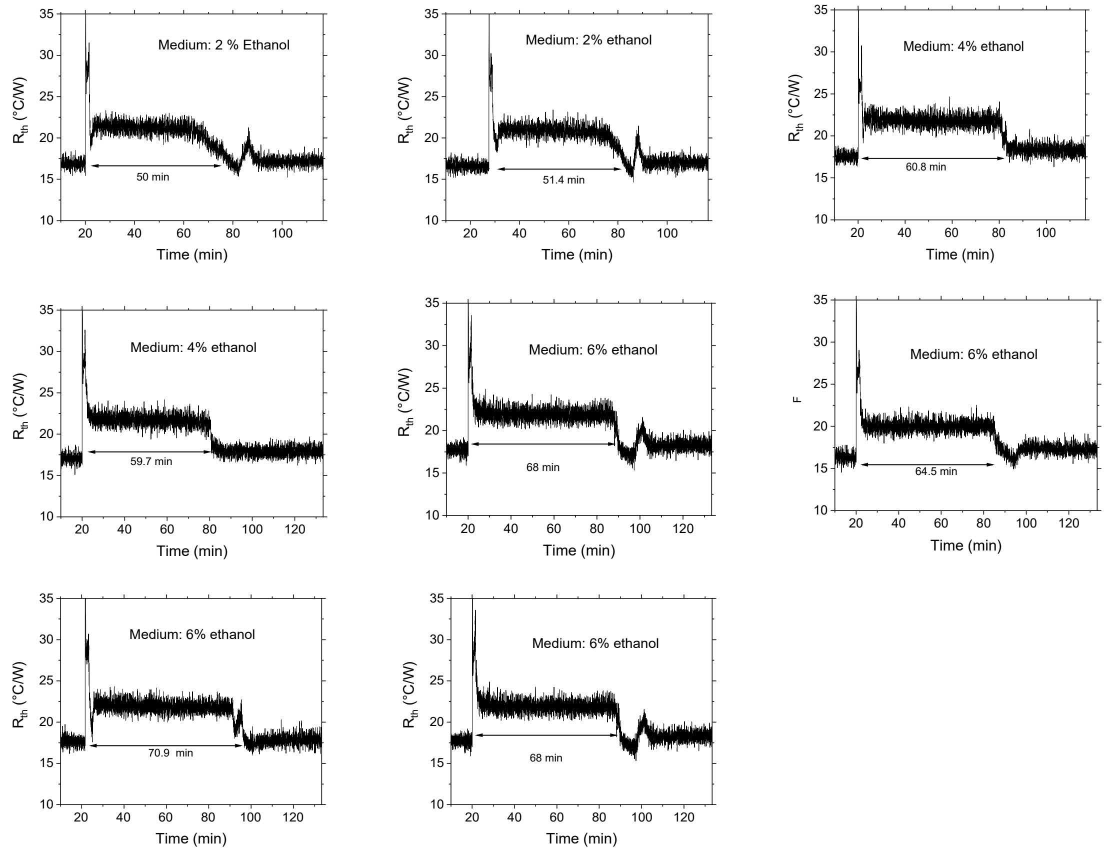

Figure S2: *Spontaneous yeast response to 2, 4 and 6% ethanol. All measurements were performed at 27°C.*

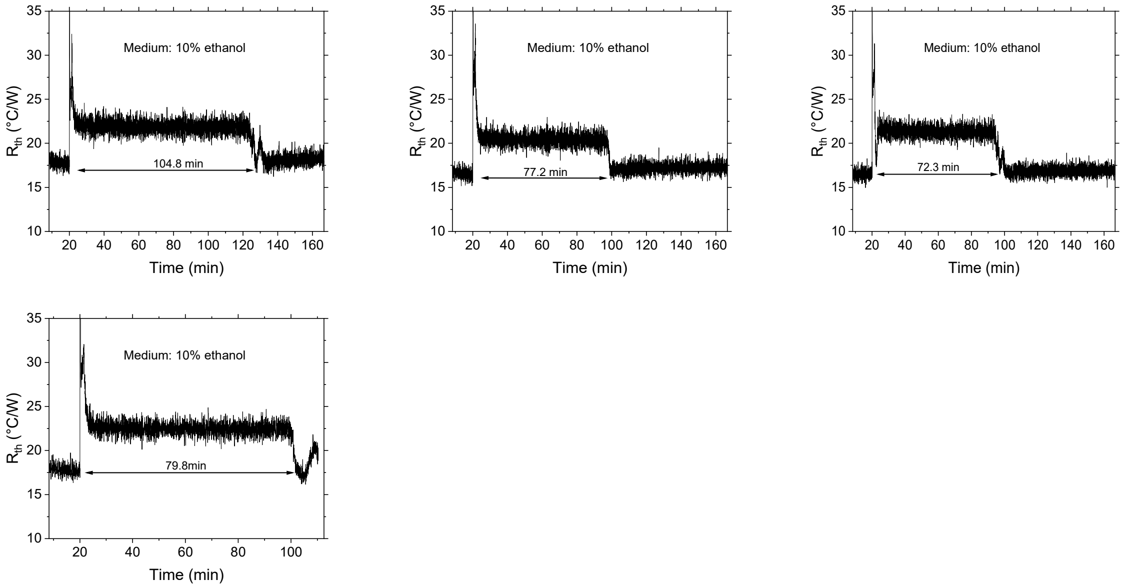

Figure S3: *Yeast response to 10% ethanol. All measurements were performed at 27°C.*

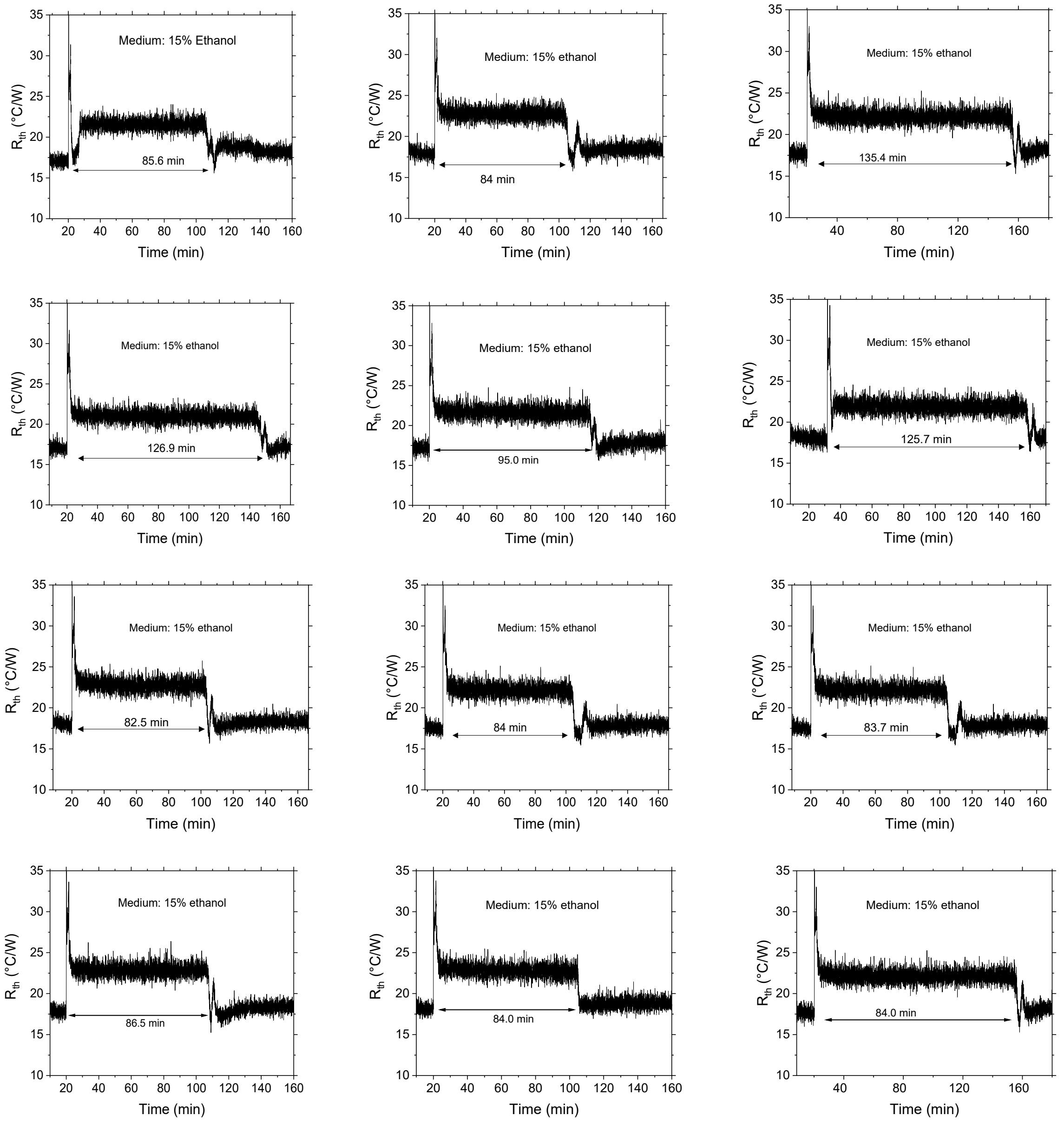

***Figure S4**: Spontaneous yeast response to 15% ethanol. The average detachment time is 100.6 ± 22.4 min. All measurements were performed at 27°C.*

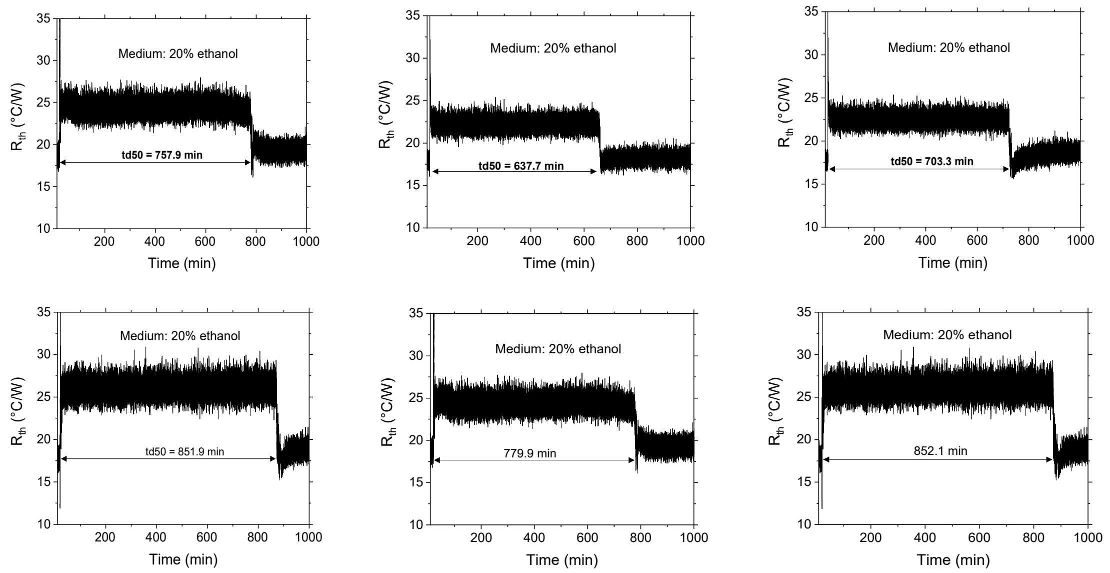

Figure S5*: Spontaneous yeast response to 20% ethanol. All measurements were performed at 27°C. Detachment times range from 638 min to 852 min with an average value of 764 ± 77 min.*

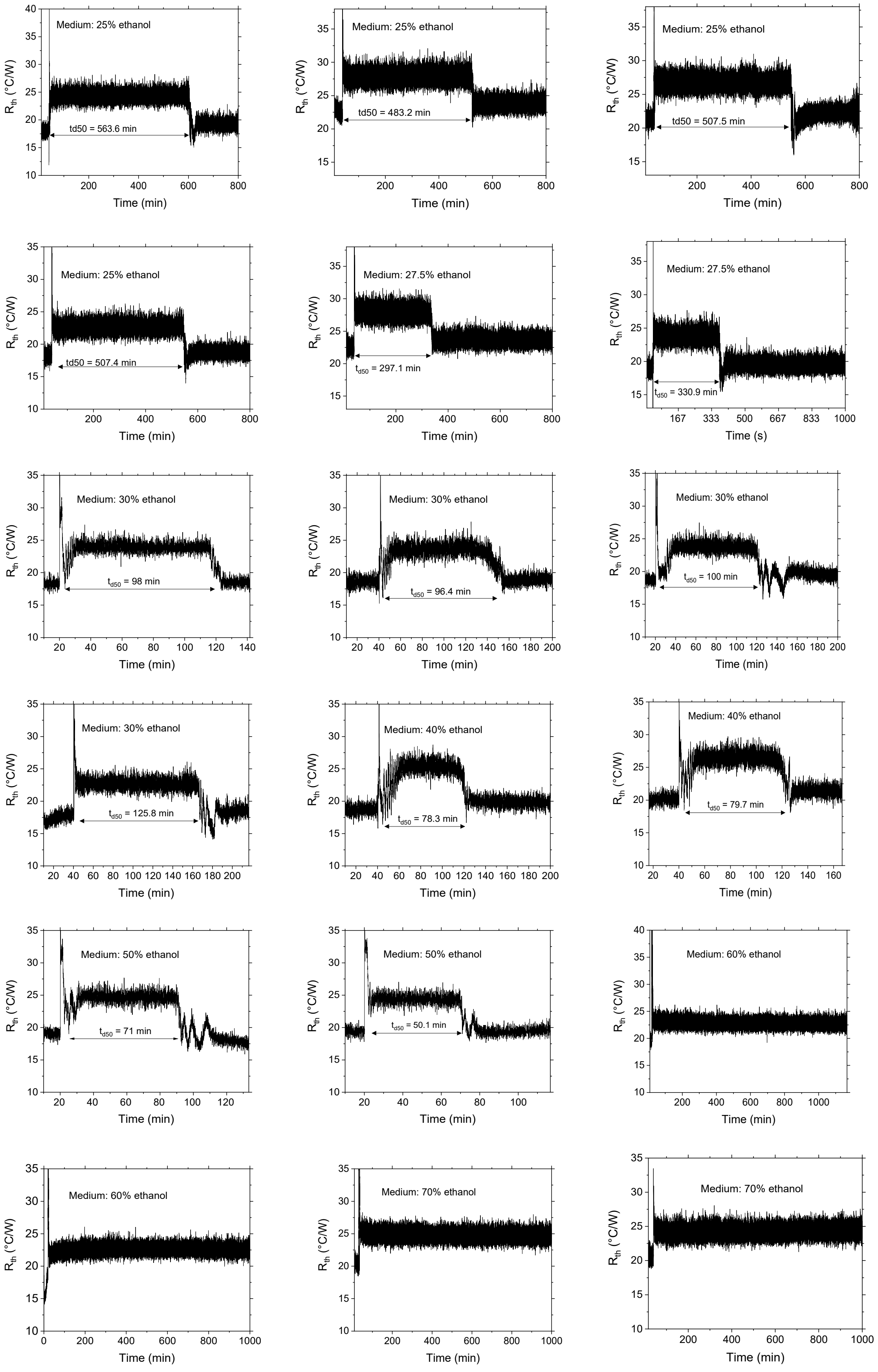

Figure S6: *Spontaneous yeast response to 25 - 70% ethanol.*

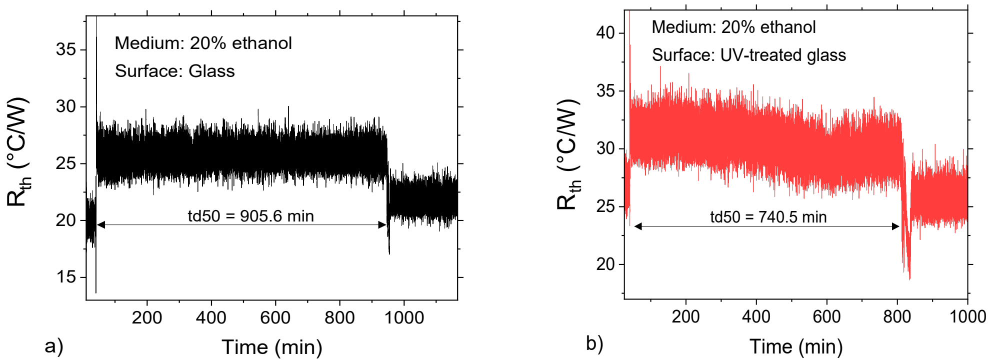

***Figure S7**: Additional measurements showing the spontaneous yeast response to 20% ethanol on glass and uv-treated glass. The detachment times lie within the values measured for pure ethanol on polyurethane-coated aluminium chips. All measurements were performed at 27°C.*

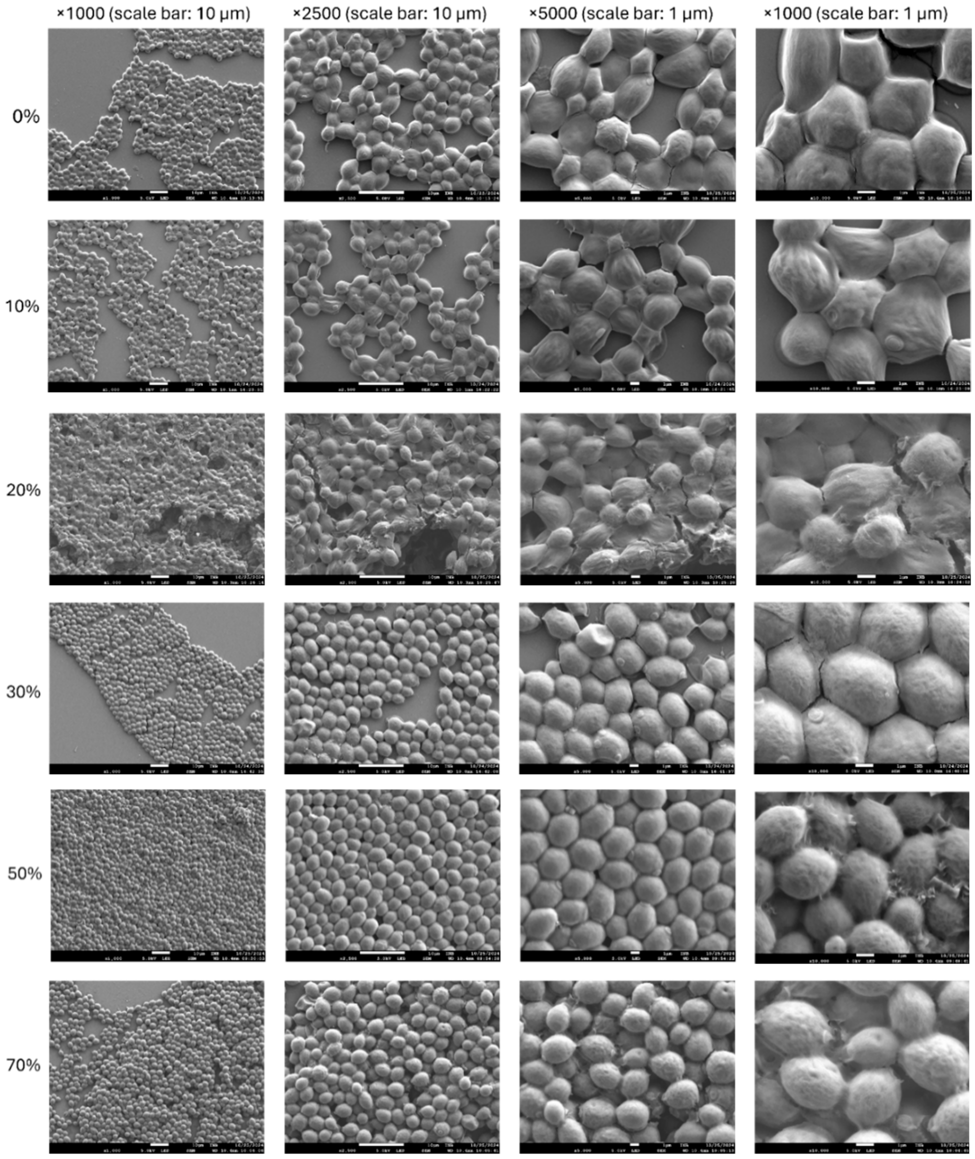

***Figure S8:** SEM analysis of yeast as a function of ethanol concentration. Cell shape transitions from slightly elongated for ≤ 20% ethanol levels to spherical for ≥ 30% ethanol.* At low ethanol levels (0–20%), cells exhibited an elongated morphology and a more spherical morphology for ethanol concentrations ≥ 30%.

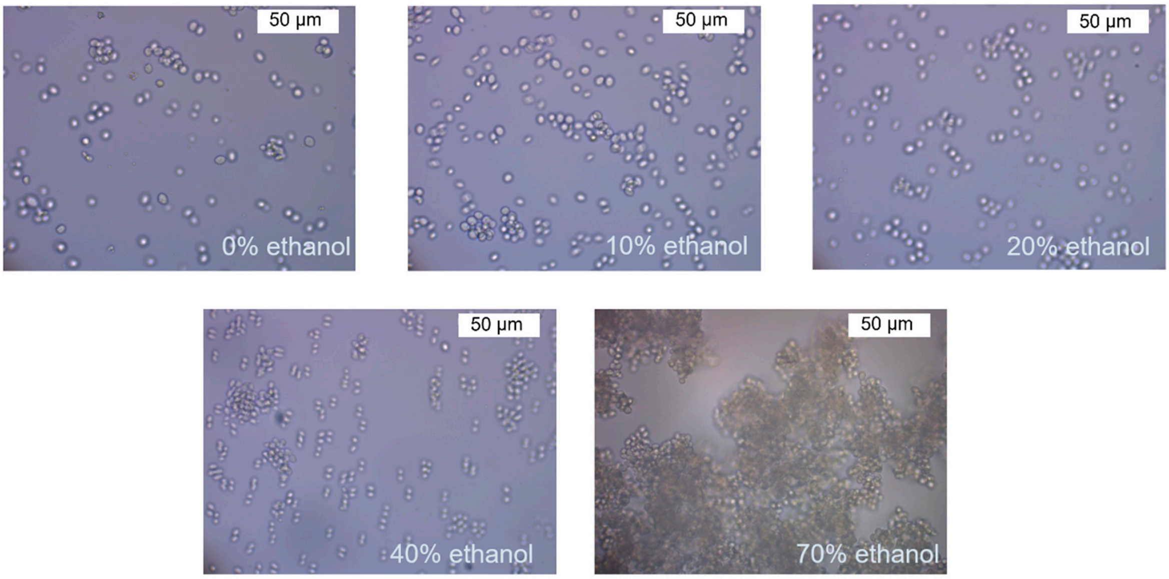

Figure S9: *Optical microscopy and dynamic OD600 analysis of cells compared by ethanol concentration. a) Optical micrographs of cells on a glass slide as a function of ethanol showing agglomeration at 70%. b)*

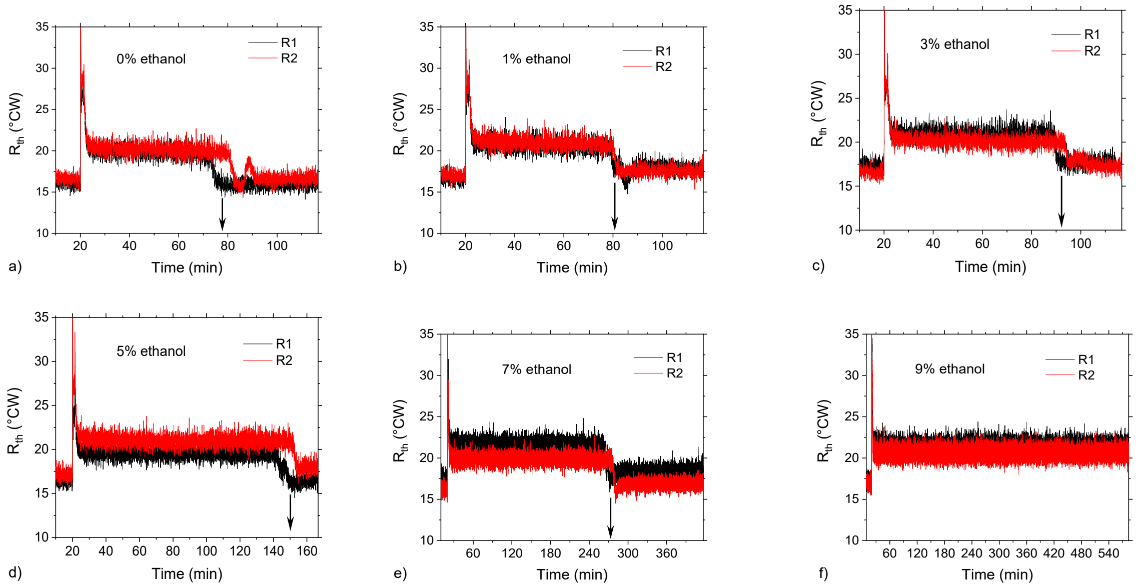

Figure S10: *Influence of ethanol on the spontaneous detachment of aged yeast cells.* ***a-f)*** *Raw data of the time-dependent heat transfer resistance (R_{th}) plots showing cell detachment at various ethanol concentrations for two independently repeated measurements.*
